# Multiplexed Molecular Imaging of Biomarker-Targeted SERS Nanoparticles on Fresh Tissue Specimens with Channel-Compressed Spectrometry

**DOI:** 10.1371/journal.pone.0163473

**Published:** 2016-09-29

**Authors:** Soyoung Kang, Yu Wang, Nicholas P. Reder, Jonathan T. C. Liu

**Affiliations:** 1 Department of Mechanical Engineering, University of Washington, Seattle, WA, United States of America; 2 Department of Pathology, University of Washington Medical Center, Seattle, WA, United States of America; Advanced Centre for Treatment Research and Education in Cancer, INDIA

## Abstract

Biomarker-targeted surface-enhanced Raman scattering (SERS) nanoparticles (NPs) have been explored as a viable option for targeting and imaging multiple cell-surface protein biomarkers of cancer. While it has been demonstrated that this Raman-encoded molecular imaging (REMI) technology may potentially be used to guide tumor-resection procedures, the REMI strategy would benefit from further improvements in imaging speed. Previous implementations of REMI have utilized 1024 spectral channels (camera pixels) in a commercial spectroscopic CCD to detect the spectral signals from multiplexed SERS NPs, a strategy that enables accurate demultiplexing of the relative concentration of each NP “flavor” within a mixture. Here, we investigate the ability to significantly reduce the number of spectral-collection channels while maintaining accurate imaging and demultiplexing of up to five SERS NP flavors, a strategy that offers the potential for improved imaging speed and/or detection sensitivity in future systems. This strategy was optimized by analyzing the linearity of five multiplexed flavors of SERS NPs topically applied on tissues. The accuracy of this binning approach was then validated by staining tumor xenografts and human breast tumor specimens with a mixture of five NP flavors (four targeted NPs and one untargeted NP) and performing ratiometric imaging of specific vs. nonspecific NP accumulation. We demonstrate that with channel-compressed spectrometry using as few as 16 channels, it is possible to perform REMI with five NP flavors, with < 20% error, at low concentrations (< 10 pM) that are relevant for clinical applications.

## 1 Introduction

Tissue-conserving surgeries, in which removal of normal tissue is minimized while attempting to achieve total resection of the tumor, is a primary intervention for many cancers, including early-stage breast cancer, early-stage colon cancer, early-stage non-small-cell lung cancer, and melanoma [1]. Numerous studies have shown that the extent of resection correlates with patient outcomes [2, 3], and that the status of the surgical margins, in particular, is of critical importance [4–6]. If a positive margin is identified on excised surgical specimens by a surgical pathologist, a re-excision procedure is often necessary to remove the residual tumor from the patient. The need for multiple surgeries burdens the healthcare system with potentially avoidable costs, while also increasing surgical risks and emotional trauma for the patient, with inferior outcomes [7–9]. Therefore, there is a need to accurately and rapidly identify tumors at surgical margin surfaces in order to guide resection procedures intraoperatively.

The molecular imaging of cell-surface proteins that are overexpressed by tumors has the potential to enable the detection of tumors with a high degree of specificity [10–12]. However, disease biomarkers vary greatly between patients, within a tumor over time, as well as at different locations within a tumor mass [13–15]. Therefore, in order to detect cancers with a high degree of sensitivity, exogenous probes should ideally be capable of being multiplexed to simultaneously image a diverse panel of disease-related biomarkers. In recent years, surface-enhanced Raman scattering (SERS) nanoparticles (NPs) have attracted interest due to their brightness, photostability, and especially their multiplexing capability with laser illumination at a single wavelength [16–20]. Different “flavors” of SERS NPs, each targeted to a unique biomarker, may be multiplexed to simultaneously image a panel of protein targets, in which demultiplexing is achieved based on the characteristic Raman fingerprint spectrum that identifies each NP flavor. It has been shown that the imaging of SERS NPs has the potential to identify phenotypically diverse tumors and to distinguish them from nonmalignant tissue [21, 22].

We have previously demonstrated that multiplexed SERS NPs may be topically applied on fresh tissue surfaces for just 5 minutes, followed by a rapid rinse-removal step (10 sec), to enable the simultaneous quantification of multiple biomarkers via raster-scanned spectral imaging with illumination from a single laser source [23–25]. The topical delivery of SERS NPs on resected tissues is attractive because it circumvents potential patient safety issues and regulatory complications associated with the *in vivo* delivery of contrast agents. A long-standing challenge with the use of exogenous contrast agents for molecular diagnostics is the presence of nonspecific and misleading sources of contrast. Example sources of nonspecific contrast include the uneven topical application and rinse removal of NPs, variable working distances of imaging probes, and variable tissue optical properties [22, 24, 26]. Another confounding factor is that differences in diffusion and passive retention exhibited by different tissues types (e.g. tumor vs. normal) can result in differences in the accumulation of topically applied NPs in those different tissues types [22–25, 27–29]. However, by simultaneously delivering one untargeted NP flavor to control for the nonspecific behavior of one or more biomarker-targeted NP flavors, a calibrated ratiometric image of specific vs. nonspecific binding can be generated to clearly identify molecularly specific NP accumulation, and therefore biomarker expression levels, for the purposes of differentiating between tumors and normal tissues [21, 22, 25, 27, 29].

**Fig 1:**
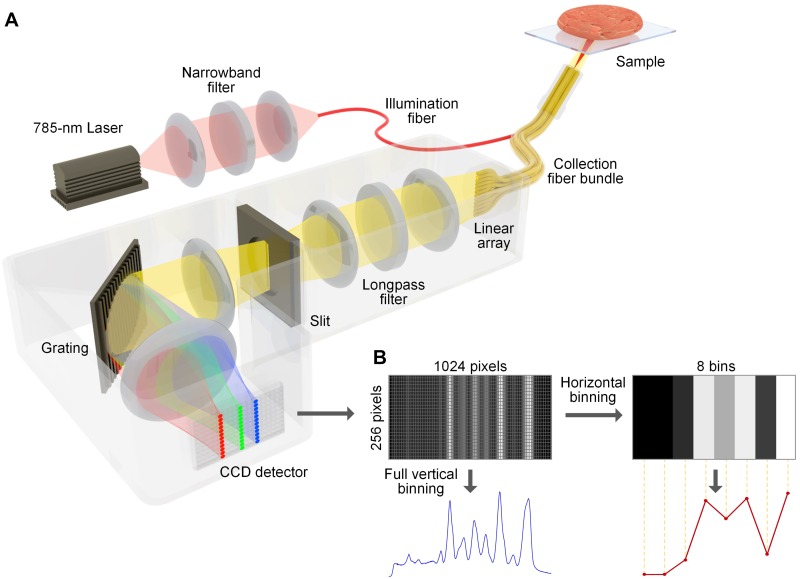
(A) The REMI system and (B) the channel-compression strategy. (**A**) Schematic of the spectral-imaging system. A 785-nm laser is used to illuminate the NP-stained tissue, creating a 1 mm-diameter laser spot. Raman-scattered photons from illuminated NPs are collected by 27 multimode fibers and transmitted to a customized spectrometer, where they are dispersed onto a cooled deep-depletion spectroscopic CCD. (**B**) The camera is used in full-vertical binning (FVB) mode, in which the signal from all 27 collection fibers are binned together by the camera, with 1024 pixels (spectral channels) used to resolve the grating-dispersed wavelength axis. In this study, the 1024-channel data set is further binned along the wavelength axis (horizontal binning) to examine the effects of spectral compression.

In order to guide tumor-resection procedures, an intraoperative imaging system should ideally provide results within a matter of minutes or seconds to minimize surgical delays and complications due to prolonged anesthesia [30]. Here, we explore the use of channel-compressed spectrometry as a potential means to improve the imaging speed of the REMI approach. The current REMI system utilizes a commercial 1024 × 256-pixel CCD operating in full-vertical-binning (FVB) mode as the detector for a spectrograph, which results in 1024 spectral channels being used for detection along the wavelength axis (see Fig 1B). One potential means of improving detection speed is to decrease the spectral resolution of our system since the frame rate of most detector-array technologies scales inversely with the number of pixels. In this study, we explore the feasibility of channel-compressed spectrometry for the detection of up to 5 multiplexed flavors of SERS NPs. Initial characterization and optimization of the channel-compression strategy is performed by analyzing the linearity of detection for 5 multiplexed flavors of SERS NPs over a range of concentrations. After determining the minimum number of spectral channels that are required for accurate detection of clinically relevant concentrations of NPs, this compression method is validated by acquiring realistic multiplexed molecular images of tumor xenografts and freshly excised human breast tumor specimens.

## 2 Materials and Methods

### 2.1 Raman-encoded molecular imaging (REMI) approach

#### 2.1.1 REMI device

A schematic of the customized REMI device is shown in Fig 1A, which has been described in detail previously [23, 24, 31, 32]. A 785-nm diode laser (∼10 mW) is used to illuminate the sample via a multimode fiber (100-μm core). The NP samples at the tissue surface are imaged at a fixed 2-mm working distance (∼1 mm illumination spot). Raman-scattered photons from illuminated SERS NPs, as well as background light (e.g. tissue autofluorescence and stray light), are collected by 27 multimode fibers (200-μm core) that surround the illumination fiber. These collection fibers are arranged in a vertical array at the entrance slit of a Raman spectrometer (Andor HoloSpec), which disperses the collected light onto a cooled deep-depletion spectroscopic CCD array (Andor, Newton DU920P-BR-DD) with 1024 × 256 pixels. The CCD is used in full-vertical-binning (FVB) mode (Fig 1B), in which the signal from all of the collection fibers are binned in the 256-pixel vertical dimension, with 1024 pixels (spectral channels) used to resolve the signal in the wavelength-dispersed dimension. The spectrometer disperses the collected light onto the CCD such that a wavelength range of 808—926 nm (Raman shift of 363—1940 cm^-1^) is examined. The detector integration time in this study is 0.1 sec (10 spectra/sec). To accomplish raster scanning, a two-axis translation stage, constructed by orthogonally assembling two linear stages (Newmark systems Inc., ET-50-11), is actuated by a two-axis stepper-motor controller (Newmark systems Inc., NSC-A2L) that is programmed in LabVIEW. Raster-scanned spectral imaging of the sample is performed by scanning the tissue sample while keeping the fiber-bundle imaging probe stationary. Since the spectral acquisition rate is 0.1 sec/spectra (0.1 sec/pixel), and the desired spatial resolution is 1 mm (Nyquist sampling pitch of 0.5 mm/pixel), the tissue is translated at a velocity of 5 mm/sec.

#### 2.1.2 Demultiplexing SERS spectra using direct classical least-squares (DCLS) fitting

The relative nanoparticle weights and the weights of all broadband background components are computed using a direct classical least-squares (DCLS) demultiplexing method described previously [20, 24, 25]. The background signals are typically broadband, unlike the narrow spectral peaks from the SERS NPs, and are due to tissue autofluorescence, photons that leak through the Raman longpass filter, and other stray light sources. The demultiplexing method is based on the assumption that the acquired spectra are linear combinations of the reference spectral components (measured beforehand). The demultiplexing is performed using the least squares function (*LSQR*) in MATLAB with a tolerance of 1 × 10^−8^ and a maximum of 500 iterations. The NP concentrations are calculated based on calibration measurements with pure (unmixed) NP flavors of known concentrations.

#### 2.1.3 SERS NPs and functionalization

SERS NPs were purchased from BD (Becton, Dickinson and Company). These NPs consist of a 60-nm diameter gold core, a layer of Raman reporters adsorbed onto the surface of the gold cores, surrounded by a 30-nm thick silica coating, resulting in an overall diameter of ∼120 nm. The five “flavors” of NPs used here are identified as S420, S421, S440, S481, and S493, and each of these emits a characteristic Raman spectrum due to chemical differences in the Raman reporter layer. Linearity measurements of the REMI system were performed with a 5-flavor mixture of these SERS NPs mixed in ratios of 1:1:1:1:1 and 3:2:1:1:1 (S420:S421:S440:S481:S493 for both NP mixtures), which reflect realistic ratio values observed in previous REMI experiments [23–25, 31]. Both NP mixtures were diluted to concentrations ranging from 0.5 to 100 pM. Additional details about the SERS NPs are available in the literature [19, 33].

For targeted molecular imaging of tissue, SERS NPs were functionalized with monoclonal antibodies (mAb) to target either the epidermal growth factor receptor (EGFR), human epidermal growth factor receptor 2 (HER2), or cluster-of-differentiation markers, CD24 and CD44, using a published conjugation protocol [23–25, 31]. Negative-control NPs were prepared by conjugating one flavor of NP to an isotype control antibody (mouse IgG1). Previous studies have shown that the isotype NP is a highly accurate control for the nonspecific behavior of the targeted NPs [24]. In brief, the NPs, which contain reactive thiols at their surface, were first reacted with a fluorophore, Cyto 647-maleamide (Cytodiagnostics Inc, part No. NF647-3-01), for the purposes of fluorescence-based flow-cytometry characterization experiments. Then, the NPs were conjugated with either an isotype control (Thermo Scientific, MA110407), an anti-EGFR (Thermo Scientific MS-378-PABX), an anti-HER2 (Thermo Scientific, MS-229-PABX), an anti-CD44 (Abcam plc., ab6124), or an anti-CD24 (Abcam plc., ab31622) monoclonal antibody (mAb) at 500 molar equivalents per NP. The NP conjugates were stored at 4-deg C and protected from light before use. UV-VIS spectrophotometry (Agilent 8453) was used to measure the concentration of the NP conjugates. Previous flow cytometry experiments [23–25, 31] have demonstrated robust binding of the EGFR-NPs and HER2-NPs to A431 cells, which highly overexpress EGFR and moderately overexpress HER2. Previous studies have also demonstrated the agreement between immunohistochemistry images (fixed tissue sections) and ratiometric REMI (fresh tissues) for quantifying the expression levels of EGFR and HER2 at the surfaces of fresh tissues [23–25, 31].

#### 2.1.4 Linearity measurements

For testing the linearity of NP detection using the REMI device, male Fischer 344 inbred rats (10 weeks, Harlan Laboratories, Inc.) were euthanized via inhalation of CO_2_, followed by the surgical removal of 2 × 3 cm^2^ sections of normal muscle tissue. After gently rinsing the tissue samples in phosphate buffered saline (PBS) and placing them on a glass slide, a 2-μL drop of either the 1:1:1:1:1 or the 3:2:1:1:1 NP mixture was placed on the surface of the rat tissue (at varying concentrations) and imaged using the REMI system. All animal work was approved by the Institutional Animal Care and Use Committee (IACUC, No. 4345-01) at the University of Washington.

### 2.2 Spectral binning

In order to explore the feasibility of channel compression in this study, a spectral-binning strategy was employed, in which the full 1024 spectral pixels collected by the commercial CCD were divided into “bins,” where the value of each bin was the mean of all pixel values within that bin (Fig 1B). We explored the variability in the demultiplexing output when the relative positions of the bins were shifted (“phase-shifted”). While the shape of the spectra varies as a function of phase (bin locations), it was determined that in terms of the accuracy of demultiplexing, the effect of phase shifting did not dominate over random experimental noise. This is because using a larger number of bins, in which the spectral features are well-resolved, allows for accurate demultiplexing regardless of phase (<5% error for 16 or more bins), while using a smaller number of bins, which causes significant blurring of the spectral features, results in low demultiplexing accuracy regardless of phase (e.g. 90% error for 8 bins). These two extreme cases illustrate why, in general, the phase of the bins does not play a major role in the accuracy of demultiplexing (as observed in our experiments for 8, 16, and 32 bins). Therefore, we disregarded phase-shifting effects in this study.

### 2.3 REMI of fresh tissues

#### 2.3.1 REMI of tumor xenografts

For validation studies, nude mice (Taconic Farms Inc, model NCRNU-F) were used to develop tumor xenografts. The A431 cancer cell line (1 × 10^6^) was suspended in matrigel (BD biosciences, 354234) in a 1:1 volume ratio to form a 200-μL mixture. At 7-9 weeks of age, nude mice were subcutaneously implanted with the cell mixture (1 × 10^6^ cells per injection) on their flanks. After 2-4 weeks, when the tumors reached a size of 8-10 mm, the mice were euthanized by CO_2_ inhalation, followed by the surgical removal of the implanted tumors. The tumor xenograft was immersed into a 50-μL equimolar mixture of the four targeted-NPs and one untargeted-NP (150 pM/flavor), and allowed to incubate for 10 min. The tissue was then submerged into 20 mL of PBS (with gentle agitation) for 10 sec to rinse off unbound NPs, followed by raster-scanned spectral imaging. For the imaging of tissue specimens, 400 spectra/cm^2^ were acquired at a spectral acquisition rate of 10 spectra/sec, thereby requiring a total imaging time of < 1 min/cm^2^. A 2-μL droplet of the original equimolar staining mixture was placed on the imaging slide near the stained tissue to calibrate the concentration and ratio measurements. After imaging, all tissues were fixed with 10% formalin and submitted for histopathology (H&E staining).

#### 2.3.2 REMI of human breast tissues

For further validation studies on human tissue, de-identified human breast tissue specimens (∼ 2 × 2 cm^2^) were obtained from the University of Washington Medical Center with patient consent and imaged within 1 hour after lumpectomy. The specimens were stained and imaged using the same protocol as was performed for REMI of tumor xenografts. Human-tissue imaging experiments were carried out in accordance with approved guidelines and all experimental protocols were approved by the Human Subjects Division at the University of Washington and the Northwest BioTrust under an IRB exemption for the acquisition of de-identified tissues. Written consent to participate in this study was provided by the patients. After imaging, specimens were fixed with 10% formalin and submitted for histopathology (H&E staining).

## 3 Results

**Fig 2:**
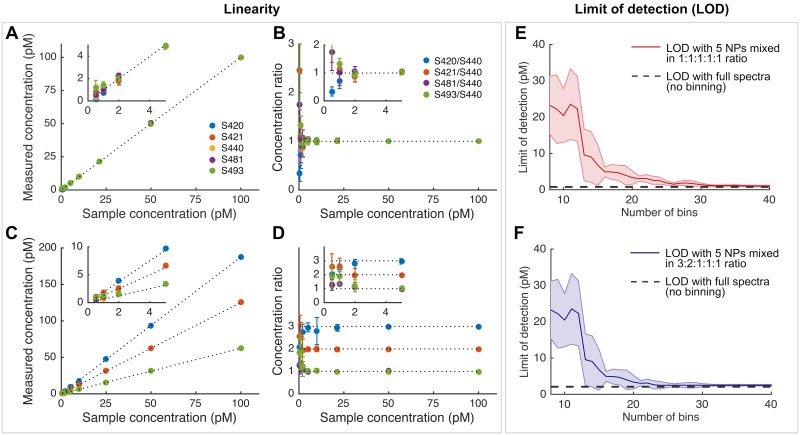
(A-D) Linearity test for 5-flavor NP mixtures and (E,F) the limit of detection (LOD) as the number of bins (spectral channels) is reduced. (**A-D**) Five NP flavors were mixed in an equimolar concentration ratio (1:1:1:1:1) or a concentration ratio of 3:2:1:1:1 (S420:S421:S440:S481:S493). Various dilutions of these NP mixtures were prepared in the range of 0.5-100 pM. The NP concentrations were measured with the REMI system to calculate their concentration ratios. (**a,b**) Linearity plots for the 1:1:1:1:1 NP mixture. (**C,D**) Linearity plots for the 3:2:1:1:1 NP mixture. Error bars represent the standard deviation across 5 repeated experiments. Note that the “sample concentration” refers to the concentration of the S440 NP (which serves as the negative-control NP). (**E,F**) The limit of detection (LOD) is defined as the concentration at which the error in the measured concentration ratio exceeds 10%. The shaded curves indicate the standard deviation in the LOD measurements from 5 repeated experiments.

The linearity of the REMI system was measured using a mixture of 5 NP flavors prepared in a concentration ratio of 1:1:1:1:1 or 3:2:1:1:1 with droplets from each mixture placed on the surface of rat tissue at varying concentrations (0.5-100 pM). Fig 2A–2D show the linearity of measured NP concentrations and concentration ratios for 5 NP flavors mixed in the 1:1:1:1:1 ratio (Fig 2A and 2B) or in the 3:2:1:1:1 ratio (Fig 2C and 2D). The concentration ratios are based on the concentration of NP flavors S420, S421, S481 and S493 against the concentration of NP flavor S440 (assumed to be the “negative-control” NP in this case). Fig 2B shows that the concentration ratios measured from the 1:1:1:1:1 NP mixtures remain near unity for NP concentrations ranging from 2-100 pM. However, for samples with NP concentrations below 1 pM, larger errors in the concentration ratio (> 10%) appear. Similarly, Fig 2D shows that the measured ratios are accurate (< 10% error) when all NP concentrations are greater than 2 pM. The non-equimolar volume ratio of 3:2:1:1:1 represents a scenario in *ex vivo* tissue imaging in which the specimen greatly overexpresses a certain protein biomarker (3-fold enhanced binding compared to the control NP), while moderately overexpressing another protein biomarker (2-fold enhanced binding compared to a control NP), with two other biomarkers that are negligibly expressed (no enhanced binding, which yields a NP ratio of 1). This range of ratios is consistent with our previous REMI experiments with fresh tissues [24, 25, 31].

A limit-of-detection (LOD) analysis was conducted using the linearity data to determine the minimal number of spectral channels needed to ensure accurate measurement of NP concentrations. In this case, the LOD is defined as the NP concentration at which the error in the concentration ratio exceeds 10%. A plot of the LOD as a function of number of spectral bins is shown in Fig 2E and 2F. These plots show that the LOD gradually deteriorates as the number of spectral channels (or bins) decreases, with the shaded region indicating the experimental variability in the LOD measurements (standard deviation, n = 5). Based on Fig 2E and 2F, the LOD remains below 10 pM as long as a minimum of 16 bins is used. Note that in REMI experiments with fresh tissues, using an optimized topical staining protocol (150 pM/flavor, 10 min of staining), the measured NP concentrations are always above 10 pM [26]. Therefore, even with only 16 spectral channels, we would expect accurate REMI measurements of tissues.

**Fig 3:**
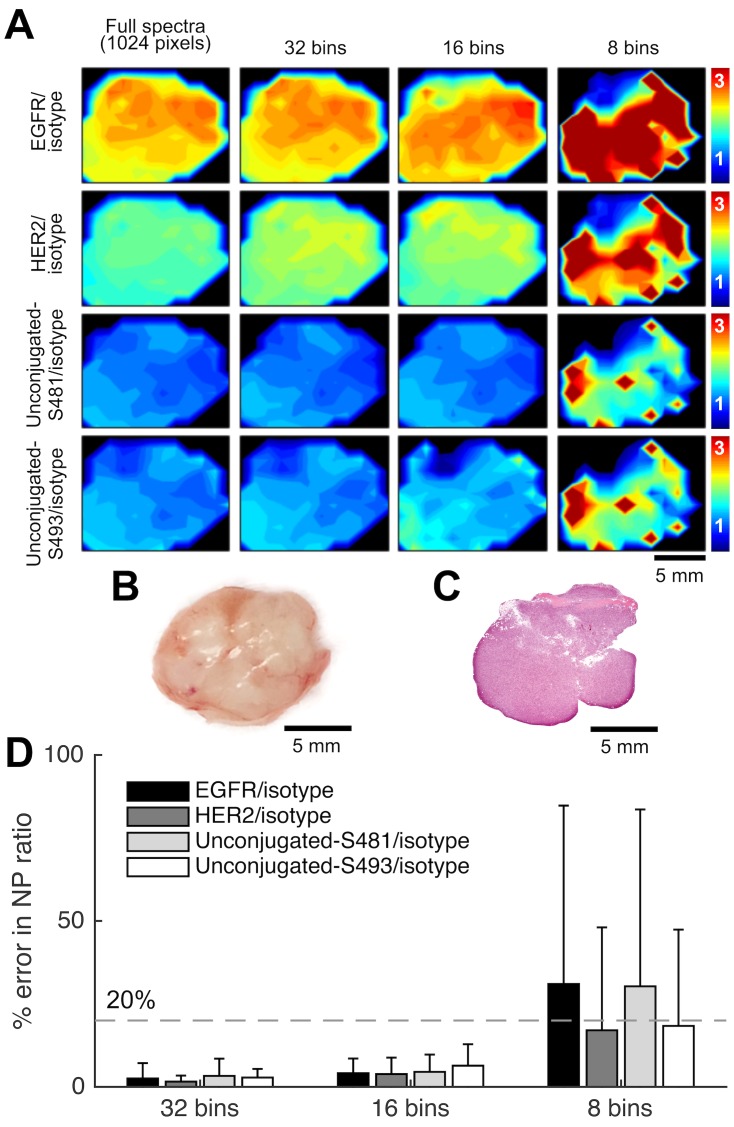
REMI approach performed on an A431 tumor xenograft (EGFR++, HER2+) stained with a 5-flavor NP mixture (EGFR-, HER2-, Control-S481-, Control-S493-, and isotype-NPs, 150 pM/flavor). (**A**) Ratiometric images of the tumor xenograft. From top to bottom, each row shows the ratio of EGFR/isotype-NP, HER2/isotype-NP, unconjugated-S481/isotype-NP, and unconjugated-S493/isotype-NP, respectively. From left to right, each column shows the ratiometric image obtained with a decreasing number of spectral channels. (**B**) A photograph of the A431 tumor xenograft. (**C**) H&E-stained pathology section of the tumor xenograft. (**D**) Average error (%) in the measured NP ratios when using spectral compression in comparison with the gold-standard images (full 1024 channels). Error bars represent the standard deviation amongst all pixels in the image.

To validate the spectral binning strategy, we first binned spectral data acquired from REMI experiments with A431 tumor xenografts (subcutaneously implanted in mice). These tissue specimens were stained for 10 min with an equimolar mixture of targeted-NPs, unconjugated NPs, and isotype-NP, in which the unconjugated NPs exhibit similar behavior to the isotype-NP [24]. Fig 3A shows the ratiometric images of targeted-NPs vs. isotype-NPs (EGFR-NP/isotype-NP and HER2-NP/isotype-NP) and unconjugated-NPs vs. isotype-NPs (unconjugated-S481-NP/isotype-NP and unconjugated-S493/isotype-NP) in each of the respective rows. The ratio of unconjugated-NPs vs. isotype-NPs is expected to lie near unity, since none of these NP flavors is targeted to a specific cell-surface molecular biomarker. The different columns in Fig 3A show ratiometric images obtained with a decreasing number of spectral channels. The first column shows the ratiometric image of an A431 tumor xenograft when no binning is performed (the 1024-channel “gold-standard” in this work), while columns 2-4 show the ratiometric images obtained when the spectral data is compressed into 32 bins, 16 bins, and 8 bins, respectively. Fig 3A shows that 32-bin and 16-bin spectral compression yields ratiometric images that are consistent with the gold-standard images, in which there is a high ratio of EGFR-NP vs. isotype-NP (∼3), a moderate ratio of HER2-NP vs. isotype-NP (∼2), and a ratio near unity for the unconjugated-S481-NP vs. isotype-NP and unconjugated-S493-NP vs. isotype-NP. Spectral compression down to 8 bins results in low-fidelity ratiometric images. Fig 3C shows an H&E-stained *en face* histology section from the same A431 tumor xenograft imaged with REMI, which confirms the relatively homogenous distribution of tumor cells throughout the tumor xenograft specimen.

To quantitatively evaluate the spectrally binned imaging data, we computed the pixel-by-pixel error of the binned images in comparison to the gold-standard (no binning) images. The results from all pixels (average and standard deviation) are displayed as bar plots in Fig 3D. With 16 bins, the errors are well below 20% (grey dashed line), and with 32 bins, the errors are below 10%. These results indicate that by using only 16 or 32 spectral channels, we can obtain ratiometric images with high fidelity to those obtained using the full 1024 spectral channels.

**Fig 4:**
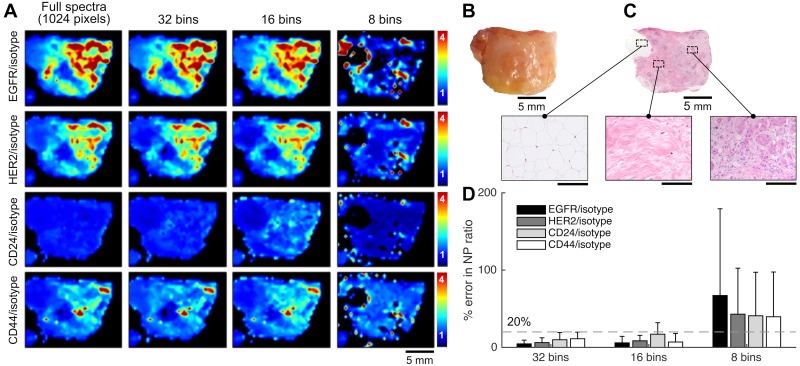
REMI approach performed on a human breast tissue specimen stained with a 5-flavor NP mixture (EGFR-, HER2-, CD24-, CD44-, and isotype-NPs, 150 pM/flavor). (**A**) Ratiometric images of a human breast tissue specimen. From top to bottom, the rows display ratiometric images of EGFR/isotype-NP, HER2/isotype-NP, CD24/isotype-NP and CD44/isotype-NP. From left to right, the columns display ratiometric images obtained with a decreasing number of spectral channels. (**B**) A photograph of the tissue specimen. (**C**) H&E histology of the specimen, with higher magnification views of fat (left), normal breast tissue (middle), and tumor (right). Unlabeled scale bars represent 200 μm. (**D**) Average error (%) in the measured NP ratios when using spectral compression in comparison to the gold-standard images (full 1024 spectral channels). The error bars represent the standard deviation amongst all pixels in the image.

The spectral binning strategy was further validated for rapid molecular imaging of fresh human breast tissues. Fig 4A shows ratiometric images of targeted-NPs vs. isotype-NPs (EGFR-NP/isotype-NP, HER2-NP/isotype-NP, CD24-NP/isotype-NP, and CD44-NP/isotype-NP) in each of the respective rows, while the columns show ratiometric images obtained with a decreasing number of spectral channels. Unlike the tumor xenograft in Fig 3, the human tissue specimen in Fig 4 exhibits a more heterogeneous tumor distribution. A high ratio of EGFR-NP vs. isotype-NP is observed (∼4), as well as a high HER2-NP vs. isotype-NP ratio (∼3.5), low CD24-NP vs. isotype-NP ratio (∼1), and a moderate CD44-NP vs. isotype-NP ratio (∼2). Qualitatively, the images obtained with 32-bin and 16-bin spectral compression exhibit image features comparable to those in the gold-standard images. The tissue was submitted for histopathology after imaging. Examination of H&E-stained sections of the tissue validated the heterogeneous distribution of the tumor cells. Fig 4D shows the error in the pixel values (average and standard deviation) between the spectrally compressed images and the gold-standard images. With only 16 bins, the errors in the ratio of EGFR-NP vs. isotype-NP, HER2-NP vs. isotype-NP and CD44-NP vs. isotype-NP are below 20% (grey dashed line). The error in the ratio of CD24-NP vs. isotype-NP is slightly worse due to the low measured concentration of CD24-NPs (∼5 pM from the poorly stained tissue regions) that was below the LOD with 16-bin spectral compression (Fig 2E and 2F). However, with 32-bin spectral compression, the errors for all NP ratios remain below 20%. While the use of 32 spectral channels may be more robust than the use of 16 spectral channels, utilizing brighter NPs and improved staining protocols may enable accurate imaging using 16 channels in the future.

## 4 Discussion and conclusions

The present study aims to explore and improve a strategy for rapid molecular imaging of superficial tissue surfaces in order to guide tumor-resection procedures. We have previously described the development of a Raman-encoded molecular imaging (REMI) system for rapid multiplexed imaging of fresh excised tissue for intraoperative guidance of breast-conserving surgery [23, 24, 31], which is the most common treatment for patients diagnosed with early-stage breast cancer [34]. The previous wide-area REMI system used 1024 spectral channels and was capable of imaging a 2 × 2 cm^2^ area of fresh tissue in approximately five minutes [23, 24]. Due to the narrow window of time that is allowed for the intraoperative assessment of the margins of excised tissue specimens, it is desired to further improve the imaging speed of REMI without compromising the accuracy in biomarker detection.

Here, we have investigated the feasibility of channel-compressed spectrometry to potentially improve the speed and/or sensitivity of REMI. We have developed and optimized a spectral binning strategy to determine the minimum number of spectral channels required to obtain ratiometric images with a desired level of accuracy when detecting low concentrations of SERS NPs. One of the primary benefits of binning is the improved signal-to-noise (SNR) ratio due to reduced read noise and increased photon counts per spectral channel/bin. The current imaging system, using a standard spectroscopic CCD, is capable of accurately demultiplexing five NP flavors down to 2 pM with < 10% errors (Fig 2A–2D). It has been determined that a minimum of 16 bins should be used to maintain the measurement accuracy when demultiplexing 5 NP flavors at a concentration of 10 pM or above. The use of 32 channels offers a LOD that is more comparable to that of 1024 channels, as well as ratiometric images with fidelity comparable to the 1024-channel “gold-standard” (Figs 3 and 4). Note that more sophisticated spectral-compression strategies are also possible, such as utilizing a limited set of spectral-detection windows that coincide with specific Raman peaks emitted by various flavors of multiplexed SERS NPs [35]. However, this would require more complex detection optics such as non-grating-based spectral filtering. On the other hand, as noted in the following paragraph, the use of uniformly spaced sequential spectral bins allows for easier adoption of certain advanced detector array technologies that are commercially available as a substitute for high-channel-count spectroscopic CCD arrays.

In summary, the results of this study are significant in showing that a very high degree of channel compression (nearly two orders of magnitude reduced from the original 1024 channels) can still allow for highly multiplexed ratiometric imaging of biomarkers with targeted SERS NPs. This opens up the possibility to use advanced detector arrays, such as a 16-channel or 32-channel photomultiplier tube (PMT) linear array or an avalanche photodiode (APD) array, which are much more sensitive and fast compared to a 1024 × 256 spectroscopic CCD array. For example, the current CCD used in our REMI system is only capable of acquiring up to 272 spectra per second in the FVB mode [36]. In comparison, PMT arrays can acquire up to 1.28 million spectra per second (4,700 times faster) due to their high cathode sensitivity and time-response, over a wide spectral range (300-920 nm) [37]. APD arrays feature comparable speed and even higher quantum efficiency compared to current PMT arrays in the 900 nm spectral range [38]. Improvements in spectral acquisition rate and SNR would not only be of great clinical benefit (through faster imaging speeds), but could also enable improved spatial resolution for REMI through the use of a smaller illumination spot size coupled with a finer sampling pitch during raster-scanned imaging (e.g. a 100-μm resolution for the identification of low numbers of tumor cells). Future studies will develop and assess the ability of a high-speed and high-resolution REMI system for a variety of clinical applications including intraoperative guidance of tumor resection and multiplexed molecular endoscopy of the gastrointestinal tract [19, 21, 23, 24, 32], in which imaging speed and imaging resolution are of importance.
